# Enhanced Anti-Cancer Effects of Conditioned Medium from Hypoxic Human Adult Dermal Fibroblasts on Cervical Cancer Cells

**DOI:** 10.3390/ijms23095134

**Published:** 2022-05-04

**Authors:** Kyu-Hyun Han, Ae-Kyeong Kim, Dong-ik Kim

**Affiliations:** Division of Vascular Surgery, Samsung Medical Center, Sungkyunkwan University School of Medicine, Seoul 06351, Korea; truehan1@empas.com (K.-H.H.); akdls@daum.net (A.-K.K.)

**Keywords:** anti-cancer, cervical cancer cells, conditioned medium, fibroblasts, hypoxic

## Abstract

Hypoxia regulates fibroblast function by changing intracellular signaling and secretion factors, that influence the states of nearby cells. In this work, we investigated how medium (CM) from human adult dermal fibroblasts (HDFs) cultured in normoxic and hypoxic conditions affected cervical cancer (HeLa) cells. The HeLa cells showed decreased cell viability, increased apoptosis, and cell cycle arrest in response to CM from hypoxic-cultured HDFs (H-CM) compared with CM from normoxic-cultured HDFs (N-CM). Among the proteins up-regulated (>2-fold) in H-CM compared with N-CM, lymphotoxin-beta receptor (LTBR) decreased the viability of HeLa cells. Among the intracellular proteins down-regulated (>2-fold) in HeLa cells treated with H-CM compared with N-CM, the most enriched biological process GO term and KEGG pathway were protein deubiquitination and hsa05166:HTLV-I infection, respectively. In the protein–protein interaction network of intracellular proteins with altered expression (>2-fold), 1 up-regulated (TNF) and 8 down-regulated (ESR1, MCL1, TBP, CD19, LCK, PCNA, CHEK1, and POLA1) hub proteins were defined. Among the down-regulated hub proteins, the most enriched biological process GO term and KEGG pathway were leading strand elongation and hsa05166:HTLV-I infection, respectively. This study reveals that H-CM had stronger anti-cancer effects on cervical cancer cells than N-CM and induced intracellular signaling patterns related to those enhanced anti-cancer effects.

## 1. Introduction

Cervical cancer is the fourth most diagnosed cancer and the fourth leading cause of cancer-related deaths in female, with approximately 604,000 new cases and 342,000 related deaths worldwide in 2020 [1]. The main treatments for cervical cancer are surgery, radiotherapy, and chemotherapy. However, recurrence after treatment occurs frequently [2,3,4], and those treatments are associated with side effects and complications [5,6,7,8]. Therefore, alternative treatments for cervical cancer with reduced side effects are needed.

Fibroblasts play an important role in the construction and repair of organs and tissues through the secretome and extracellular matrix reorganization [9]. Previous studies reported that conditioned medium (CM) from fibroblasts promoted the proliferation and differentiation of cardiomyoblasts and the adhesion activity and angiogenesis of umbilical vein endothelial cells [10,11].

When fibroblasts are exposed to pro-inflammatory cytokines such as TNFα and IL-1β, they release CXCL10 and promote the M1 phenotype polarization of macrophages [12]. In addition, in response to secretory immunoglobulin (Ig) A, fibroblasts release inflammatory mediators such as IL-6, IL-8, MCP-1, and GM-CSF, that enhance cell proliferation [13]. CM from blue light–emitting diode–exposed fibroblasts had anti-inflammatory effects on lipopolysaccharide-stimulated macrophages [14], and CM from fibroblasts treated with compressive force promoted macrophage M1 polarization [15].

Previous studies showed that hypoxic conditions change the metabolism of fibroblasts by increasing their glucose intake and lactate production, which up-regulates the HIF pathway and thereby enhances cell proliferation, migration, and viability [16,17]. However, in severely hypoxic conditions (0.1% O_2_), fibroblasts undergo cell-cycle arrest through the p53–p21 pathway [18]. In addition, hypoxic conditions increase the expression of angiogenic factors such as ANG and VEGF [19] and enhance T cell cytotoxicity by increasing the secretion of immunosuppressive factors such as TGF-β, IL-6, IL-10, and PD-L1 [20].

LTBR is a member of the TNF family of receptors [21] and activates the nuclear factor-κB signaling pathway [22]. Furthermore, LTBR is involved in cell survival [23] and cell death [24], as well as the maturation of peripheral lymphoid tissues [25]. Other studies have shown that LTBR signaling is involved in tumorigenesis, and LTBR has been considered as a target for anti-tumor therapy [26]. In contrast, studies have also reported that LTBR signaling induces tumor cell apoptosis [27,28], and the overexpression of LTBR in HeLa cells triggered ligand-independent apoptosis [29].

Cancer-associated fibroblasts (CAFs) play an important role in cancer invasion and metastasis by remodeling the extracellular matrix, modulating the epithelial-to-mesenchymal transition in cancer cells, and facilitating communication among cancer cells by means of secretion factors [30]. In contrast, one study showed that CD36+ fibroblasts and CM strongly suppressed the growth of breast cancer cells; the secretory proteins in the CM that were involved in those effects were SLIT3, FBLN1, and PENK [31]. In another study, CM from the hypoxic-cultured fibroblasts promoted the invasiveness of pancreatic cancer cells through the HGF/c-Met pathway [32]. In our previous study, treating HeLa cells with CM from the hypoxic-cultured human umbilical cord–derived mesenchymal stem cells (HUC-MSCs) strongly decreased their viability [33].

Based on those findings, we investigated whether CM from hypoxic human adult dermal fibroblasts (HDFs) (H-CM) produced anti-cancer effects in cervical cancer cells. We also profiled the secretory proteins in H-CM and compared them with those in CM from normoxic HDFs (N-CM) to investigate the anti-cancer factors. We further used a protein antibody array to determine the intracellular signaling patterns and hub proteins that were induced in HeLa cells by H-CM treatment.

## 2. Results

### 2.1. H-CM Strongly Decreased Viability and Increased Apoptosis in HeLa Cells

**Figure 1:**
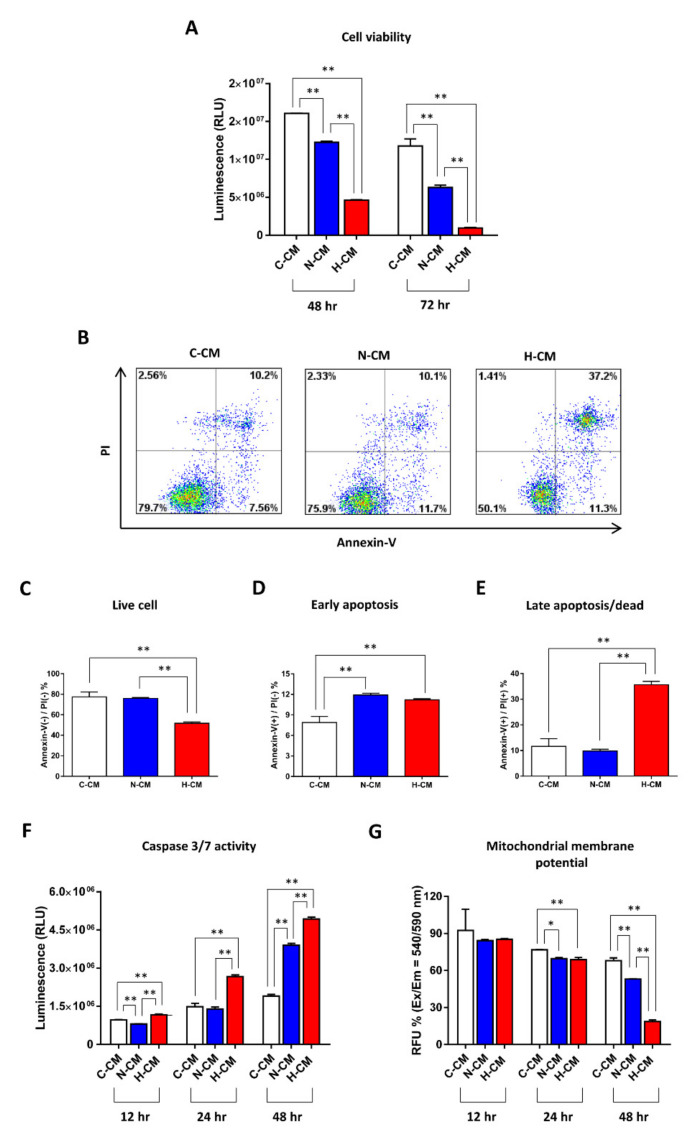
H-CM decreased the viability and increased the apoptosis of HeLa cells. The viability of HeLa cells treated with C-CM, N-CM, or H-CM (**A**). The viability of HeLa cells was measured using the CellTiter-Glo assay after 48 and 72 h of treatment with C-CM, N-CM, or H-CM. The proportion of live cells [Annexin (−)/PI(−)] (**B**,**C**), early apoptotic cells [Annexin (+)/PI(−)] (**B**,**D**), and late apoptotic cells [Annexin (+)/PI(+)] (**B**,**E**) among HeLa cells treated with C-CM, N-CM, or H-CM. Cells were evaluated using the Annexin/PI staining assay and flow cytometry after 48 h of treatment with C-CM, N-CM, or H-CM. Caspase-3/7 activity in HeLa cells treated with C-CM, N-CM, or H-CM (**F**). Caspase-3/7 activity in HeLa cells was measured using the Caspase-Glo 3/7 Assay after treatment with C-CM, N-CM, or H-CM for 12 h to 48 h. The MMP activity in HeLa cells treated with C-CM, N-CM, or H-CM (**G**). The MMP activity in HeLa cells was measured using the Orange Mitochondrial Membrane Potential Assay after treatment with C-CM, N-CM, or H-CM for 12 h to 48 h. Data are expressed as the mean ± SD of experiments performed in triplicate (each group *n* = 3, * *p* < 0.05, ** *p* < 0.01, one-way ANOVA). C-CM, serum-free medium as control; N-CM, conditioned medium from normoxic HDFs; H-CM, conditioned medium from hypoxic HDFs. Image (**B**) was produced using FlowJo v10 (Treestar, San Carlos, CA, USA). Images (**A**,**C**–**F**) were produced using GraphPad Prism version 7.00 (GraphPad Software, San Diego, CA, USA).

The study design was described with a graphic representation in Supplementary Figure S1. The ratio of cell proliferation and cell viability did not change between the normoxic and hypoxic HDFs at passage 6 (Supplementary Figure S2A,B). The viability of HeLa cells decreased more strongly after 48 h and 72 h of treatment with H-CM than it did with the serum-free medium used as the control (C-CM) and N-CM treatment (Figure 1A). The viability of other cervical cancer cells, CaSki, ME-180, and SiHa cells, also decreased with H-CM treatment compared with C-CM or N-CM treatment at 72 h (Supplementary Figure S4A–C). Other cancer cell lines, melanoma (A375SM), breast cancer (MCF-7), and lung cancer (NCI-H358) cells, also showed decreased viability after 72 h of treatment with H-CM compared with C-CM or N-CM (Supplementary Figure S3D–F). Compared with C-CM or N-CM treatment, HeLa cells treated with H-CM for 48 h had a significantly decreased proportion of live cells, as indicated by Annexin-V(−)/PI(−) (Figure 1B,C). More early apoptotic cells, as indicated by Annexin-V(+)/PI(−), were found with N-CM or H-CM treatment than with C-CM treatment (Figure 1B,D). The number of late apoptotic cells, as indicated by Annexin-V(+)/PI(+), was strongly increased by H-CM treatment compared with N-CM or C-CM treatment (Figure 1B,E). The activity of caspase-3/7, a representative apoptotic marker, increased at 12–48 h when HeLa cells were treated with H-CM, compared with C-CM or N-CM (Figure 1F). The mitochondrial membrane potential (MMP) of HeLa cells treated with N-CM or H-CM decreased compared with C-CM treatment at 24 h; only the decrease with H-CM remained strong at 48 h (Figure 1G).

### 2.2. H-CM Strongly Induced Cell Cycle Arrest in HeLa Cells

**Figure 2:**
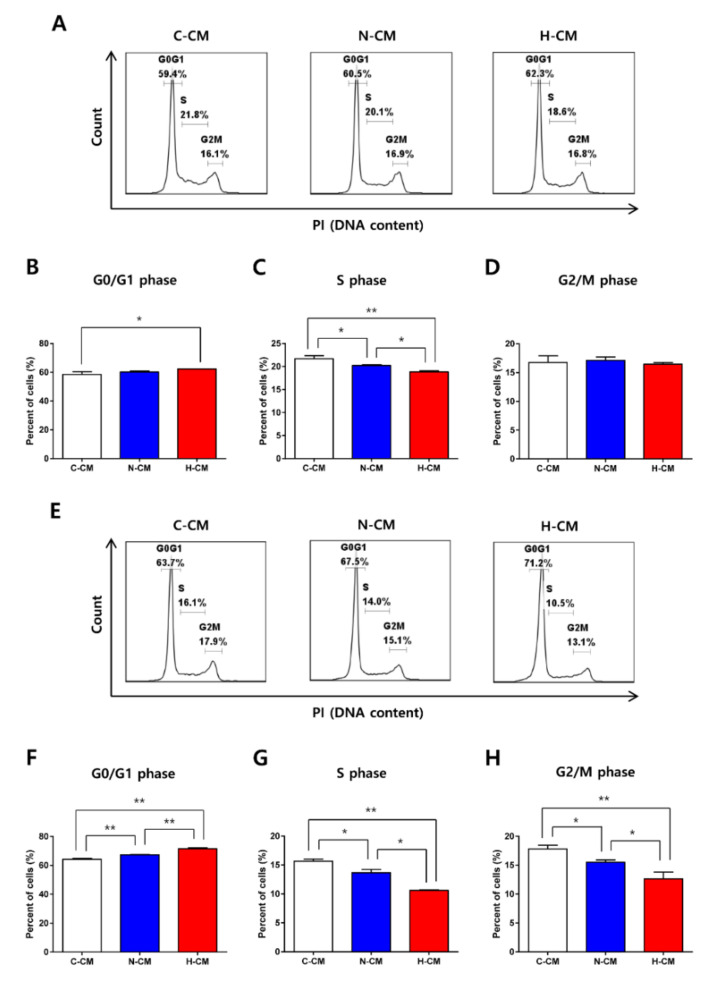
H-CM strongly induced cell cycle arrest in HeLa cells. The percentage of HeLa cells in the G0/G1 phase (**A**,**B**), S phase (**A**,**C**), and G2/M phase (**A**,**D**) after 24 h of treatment with C-CM, N-CM, or H-CM. The percentage of HeLa cells in the G0/G1 phase (**E**,**F**), S phase (**E**,**G**), and G2/M phase (**E**,**H**) treated for 48 h with C-CM, N-CM, or H-CM. The cells were incubated with C-CM, N-CM, or H-CM for 24 h or 48 h, and the cell cycle was analyzed using PI staining and flow cytometry. Data are expressed as the mean ± SD experiments performed in triplicate (each group *n* = 3, * *p* < 0.05, ** *p* < 0.01, one-way ANOVA). C-CM, serum-free medium as control; N-CM, conditioned medium from normoxic HDFs; H-CM, conditioned medium from hypoxic HDFs; PI, propidium iodide. Images (**A**,**E**) were produced using FlowJo v10 (Treestar). Images (**B**–**D**,**F**–**H**) were produced using GraphPad Prism version 7.00 (GraphPad Software).

We compared the cell cycle of HeLa cells in response to H-CM with that in response to C-CM or N-CM treatment for 24 h. The proportion of HeLa cells in the G0/G1 phase increased (Figure 2A,B) upon H-CM treatment compared with C-CM, and the proportion in the S phase decreased (Figure 2A,C) upon H-CM treatment compared with C-CM or N-CM treatment. The proportion of cells in the G2/M phase did not change significantly with the different CM treatments (Figure 2A,D). After 48 h of CM treatment, the proportion of cells in the G0/G1 phase increased (Figure 2E,F), and the proportion of cells in the S and G2/M phases decreased (Figure 2E,G,H) in the H-CM condition compared with the C-CM and N-CM conditions.

### 2.3. Profiling of Proteins Up- and down-Regulated by H-CM Compared with N-CM

**Figure 3:**
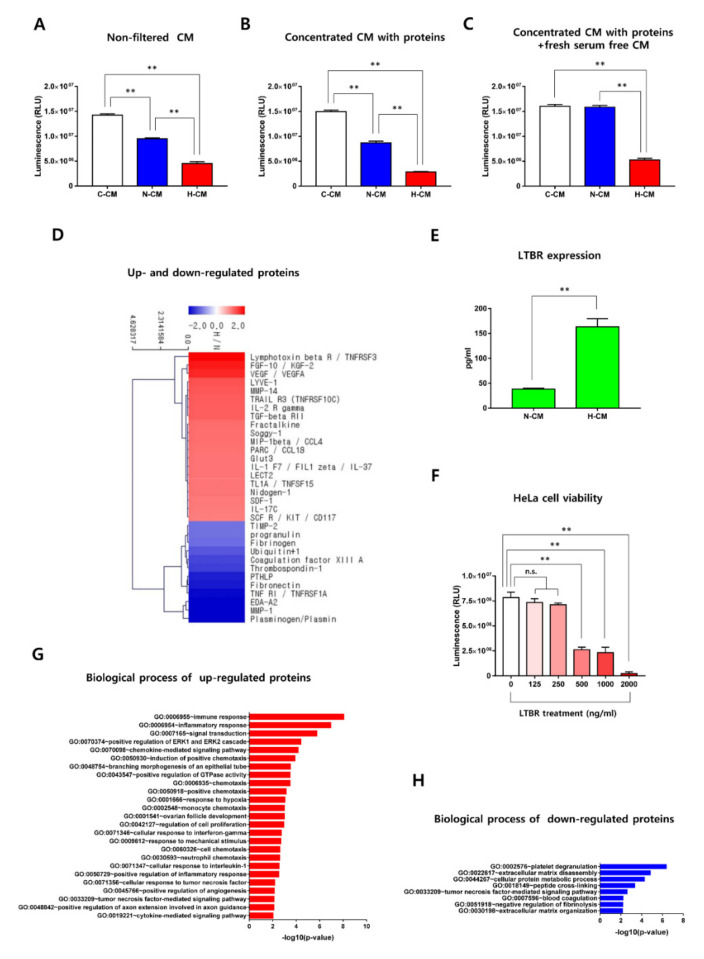
Proteins in H-CM decreased the viability of HeLa cells. The viability of HeLa cells treated with non-filtered C-CM, N-CM, or H-CM (**A**). The viability of HeLa cells treated with concentrated C-CM, N-CM, or H-CM with proteins (**B**). The viability of HeLa cells treated with concentrated C-CM, N-CM, or H-CM with proteins in fresh serum-free medium (**C**). The viability of HeLa cells was measured using the CellTiter-Glo assay after 72 h of treatment with the given CM. Data are expressed as the mean ± SD of experiments performed in triplicate (each group *n* = 3, * *p* < 0.05, ** *p* < 0.01, *t*-test). A clustering image [34] of proteins up- (red) and down-regulated (blue) (>2-fold) in H-CM compared with N-CM was produced using a protein antibody array (**D**). The image in (**D**) was produced using MeV v 4.9.0 (J.Craig Venter Institute, Rockville, USA). LTBR protein expression in N-CM and H-CM (**E**). The expression of LTBR protein in N-CM and H-CM was measured by ELISA. The viability of HeLa cells treated with recombinant LTBR protein (**F**). The cell viability was measured using a CellTiter-Glo assay after 72 h of treatment with various doses of recombinant LTBR protein. The data in (**E**,**F**) are expressed as the mean ± SD of experiments performed in triplicate (each group *n* = 3, * *p* < 0.05, ** *p* < 0.01, *t*-test, one-way ANOVA). Biological process terms from the GO analysis of proteins up-regulated in H-CM compared with N-CM (**G**). Biological process terms from the GO analysis of proteins down-regulated in H-CM compared with N-CM (**H**). The GO data are represented using the −log10 (*p*-value) (*p* < 0.01). C-CM, serum-free medium as control; N-CM, conditioned medium from normoxic HDFs; H-CM, conditioned medium from hypoxic HDFs. Images (**A**–**C**,**E**–**H**) were produced using GraphPad Prism version 7.00 (GraphPad Software).

To determine whether the proteins in CM have anti-cancer effects, we filtered and concentrated the CM. When non-filtered CM and concentrated CM were administered to HeLa cells, viability was significantly reduced by both kinds of H-CM compared with both kinds of C-CM and N-CM (Figure 3A,B). To exclude the anti-cancer effects of exhausted metabolites in the CMs, the concentrated CMs with proteins were mixed with fresh serum-free medium to the same total volume as the non-filtered CM and administered to HeLa cells. The viability of the HeLa cells was also significantly reduced by the concentrated H-CM with proteins mixed with fresh serum-free medium compared with the concentrated C-CM or N-CM with proteins mixed with fresh serum-free medium (Figure 3C).

To investigate the secretory proteins in H-CM compared with N-CM, a protein antibody array was performed. Among the 10,000 proteins in the antibody array, 20 proteins were up-regulated or down-regulated by at least two-fold in H-CM compared with N-CM (Figure 3D, Supplementary Table S1). Among the up-regulated proteins, we chose LTBR protein (which showed the highest up-regulation, 6.189-fold) (Figure 3D, Supplementary Table S1) for further investigation. The expression of LTBR in N-CM and H-CM was evaluated by ELISA, and the results showed that LTBR was more highly expressed in H-CM than N-CM (Figure 3E). When 500 ng/mL to 2000 ng/mL of recombinant LTBR protein was administered to HeLa cells for 72 h, the viability of the HeLa cells decreased significantly (Figure 3F). To categorize the proteins up- and down-regulated in H-CM, a GO analysis was performed using the Database for Annotation, Visualization, and Integrated Discovery (DAVID). The data are described using the −log10 (*p*-value) (*p* < 0.01).

Among the up-regulated proteins, the most enriched biological process GO term was immune response (8.062), followed by inflammatory response (6.948), signal transduction (5.766), positive regulation of the ERK1 and ERK2 cascade (4.408), chemokine-mediated signaling pathway (4.175), induction of positive chemotaxis (3.899), branching morphogenesis of an epithelial tube (3.520), positive regulation of GTPase activity (3.478), chemotaxis (3.478), positive chemotaxis (3.152), response to hypoxia (3.043), monocyte chemotaxis (2.993), ovarian follicle development (2.993), regulation of cell proliferation (2.951), cellular response to interferon-gamma (2.729), response to mechanical stimulus (2.700), cell chemotaxis (2.617), neutrophil chemotaxis (2.603), cellular response to interleukin-1 (2.541), positive regulation of inflammatory response (2.517), cellular response to tumor necrosis factor (2.170), positive regulation of angiogenesis (2.133), tumor necrosis factor–mediated signaling pathway (2.111), positive regulation of axon extension involved in axon guidance (2.103), and cytokine-mediated signaling pathway (2.024) (Figure 3G, Supplementary Table S2).

Among the down-regulated proteins, the biological process GO terms were platelet degranulation (6.370), extracellular matrix disassembly (4.844), cellular protein metabolic process (4.271), peptide cross-linking (3.328), tumor necrosis factor–mediated signaling pathway (2.588), blood coagulation (2.211), negative regulation of fibrinolysis (2.185), and extracellular matrix organization (2.158) (Figure 3H, Supplementary Table S3).

### 2.4. Profiling of Intracellular Proteins Induced in HeLa Cells by H-CM Compared with N-CM

**Figure 4:**
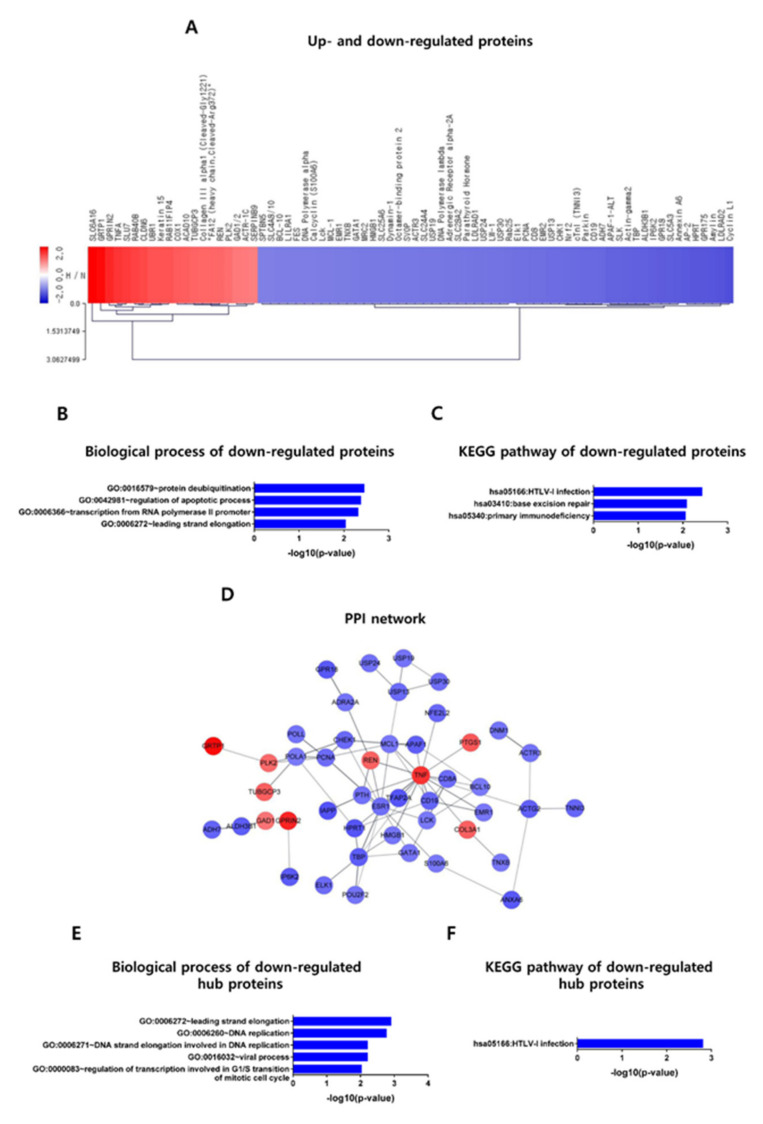
Profiling of intracellular signaling and the selection of hub proteins in HeLa cells treated with H-CM or N-CM. Intracellular proteins up- (red) and down-regulated (blue) (>2-fold) [34] in HeLa cells treated with H-CM compared with N-CM, as identified by a protein antibody array analysis (**A**). Biological process terms from the GO analysis of proteins down-regulated in HeLa cells treated with H-CM compared with N-CM (**B**). The KEGG pathway analysis [35] of proteins down-regulated in HeLa cells treated with H-CM compared with N-CM (**C**). PPI network [36,37] of intracellular proteins up- and down-regulated in HeLa cells treated with H-CM compared with N-CM (**D**). The biological process terms from the GO analysis of hub proteins down-regulated in HeLa cells treated with H-CM compared with N-CM (**E**). The KEGG pathway [35] analysis of hub proteins down-regulated in HeLa cells treated with H-CM compared with N-CM (**F**). The data for the GO and KEGG pathway results [35] are represented as the −log10 (*p*-value) (*p* < 0.01). Red, up-regulated proteins; blue, down-regulated proteins; PPI, protein–protein interaction; N-CM, conditioned medium from normoxic HDFs; H-CM, conditioned medium from hypoxic HDFs. Image in (**A**) was produced using MeV v 4.9.0 (J.Craig Venter Institute). Images in (**B**,**C**,**E**,**F**) were produced using GraphPad Prism version 7.00 (GraphPad Software). Image in (**D**) was produced using Cytoscape-STRING v 1.5.0 (NIGMS, Bethesda, MA, USA).

To investigate the intracellular signaling patterns in HeLa cells that were involved in the anti-cancer effects of H-CM and differed from those of N-CM, we performed a protein antibody array of 1,358 proteins. Compared with N-CM, H-CM up-regulated 20 proteins and down-regulated 56 proteins (>2-fold) in HeLa cells (Figure 4A, Supplementary Table S4). The proteins were categorized through a GO analysis and KEGG pathway analysis in DAVID, and the data are described using the −log10 (*p*-value). The GO biological processes and KEGG pathways were not determined for the up-regulated proteins. In the GO analysis of the down-regulated proteins (*p* < 0.01), the most enriched biological process term was protein deubiquitination (2.448), followed by regulation of the apoptotic process (2.373), transcription from RNA polymerase II promoter (2.311), and leading strand elongation (2.033) (Figure 4B, Supplementary Table S5). The KEGG pathway analysis of down-regulated proteins (*p* < 0.01) returned hsa05166:HTLV-I infection (2.422), hsa03410:base-excision repair (2.078), and hsa05340:primary immunodeficiency (2.053) (Figure 4C, Supplementary Table S5).

### 2.5. Protein–Protein Interaction (PPI) Network and Hub Protein Selection

To identify PPIs and select hub proteins differentially expressed in HeLa cells treated with H-CM compared with N-CM, the STRING database and Cytoscape 3.7.0 (NIGMS, Bethesda, USA) were used. A total of 47 nodes (proteins) and 74 edges (protein interaction lines) composed the PPIs of the intracellular proteins up- and down-regulated in HeLa cells treated with H-CM compared with those treated with N-CM (Figure 4D, Table 1). Interacting proteins with more than 5 edges in a PPI were defined as hub proteins. Using that definition, tumor necrosis factor (TNF) (18 edges, +3.002-fold), ESR1 (12 edges, −2.142-fold), MCL1 (7 edges, −2.035-fold), TBP (7 edges, −2.355-fold), CD19 (6 edges, −2.257-fold), LCK (6 edges, −2.030-fold), PCNA (6 edges, −2.172-fold), CHEK1 (5 edges, −2.205-fold), and POLA1 (5 edges, −2.022-fold) were hub proteins (Table 1). Protein expression was validated by Western blotting (Supplementary Figure S4).

GO and KEGG pathway analyses were applied to the hub proteins to determine the signal pathway patterns, and the data are described as −log10 (*p*-value). With only one up-regulated protein, TNF, the GO terms and KEGG pathways were not determined. For the 8 down-regulated hub proteins, the biological process GO terms were leading strand elongation (2.903), DNA replication (2.763), DNA strand elongation involved in DNA replication (2.205), viral process (2.204), and regulation of transcription involved in G1/S transition of the mitotic cell cycle (2.020) (Figure 4E, Table 2). The KEGG pathway analysis returned only hsa05166:HTLV-I infection (2.807) (Figure 4F, Table 2).

## 3. Discussion

This work demonstrated that H-CM treatment produced enhanced anti-cancer effects in HeLa cells compared with N-CM treatment. Furthermore, we profiled the secretory proteins in H-CM and compared them with those in N-CM. We found that LTBR, which was highly expressed in H-CM, showed anti-cancer effects, and we identified the intracellular signaling patterns and hub proteins related to the enhanced anti-cancer effects.

In a previous study, hypoxic culture conditions (1% O_2_) increased proliferation of human pulmonary fibroblasts but not cell viability for 3 and 6 days [17], whereas severe hypoxic culture conditions (0.1% O_2_) induced cell cycle arrest and moderate hypoxia (2% O_2_) enhances cell proliferation of human lung fibroblast for 24 h [18]. In our experiment, the proliferation and viability of HDFs did not increase in hypoxic culture conditions (1% O_2_) (Supplementary Figure S2A,B). These contradictory results could be caused by differences in the hypoxic culture conditions, different kinds of cells, and hypoxic culture duration.

LTBR protein, which was more highly expressed in H-CM than N-CM according to a protein antibody array and ELISA (Figure 3D,E, Supplementary Table S1), decreased the viability of HeLa cells (Figure 3F). Previous studies [27,28,29] support our results showing that the viability of HeLa cells decreased upon treatment with recombinant LTBR protein (Figure 3F). The intracellular signaling patterns associated with the anti-cancer effects of treating HeLa cells with recombinant LTBR protein will be investigated in future studies. Other studies reported that IL-37 [38], LECT2 [39], and TNFSF15 [40] suppress tumor growth. Those three proteins could support the anti-cancer effects of H-CM and serve as candidates for the development of anti-cancer drug cocktails.

The biological process GO terms for the intracellular signaling patterns of proteins down-regulated in HeLa cells treated with H-CM included protein deubiquitination, regulation of the apoptotic process, transcription from RNA polymerase II promoter, and leading strand elongation (Figure 4B, Supplementary Table S5). The most enriched term in the KEGG pathway analysis was hsa05166:HTLV-I infection, and hsa03410:base-excision repair and hsa05340:primary immunodeficiency were also enriched (Figure 4C, Supplementary Table S5). HTLV-I infection plays a role in the cellular transformation and tumorigenesis of CD4+ T-lymphocytes into adult T-cell leukemia/lymphoma cells [41]; base-excision repair induces the proliferation of prostate cancer [42]; and primary immunodeficiency is related to malignancy in patients with primary immunodeficiency disorder [43]. Therefore, those terms could be negatively regulated by H-CM because they were enriched in proteins down-regulated in HeLa cells treated with H-CM. Those terms could be thus considered as targets for effective anti-cancer therapies.

In the PPI network of proteins up- and down-regulated in HeLa cells treated with H-CM, 1 up-regulated (TNF) and 8 (ESR1, MCL1, TBP, CD19, LCK, PCNA, CHEK1, and POLA1) down-regulated hub proteins with more than 5 edges were identified (Figure 4D, Table 1, Supplementary Figure S4). TNF, the only up-regulated hub protein, functions as a tumor stimulator or suppressor [44], depending on the organ, cell, and carcinogen. Most of the down-regulated hub proteins (ESR1 [45], MCL1 [46], LCK [47], TBP [48], and PCNA [49]) play roles in the proliferation or survival of cancer cells, and CHEK1 [50] is associated with the cell cycle checkpoint in cancer cells. In addition, CD19 [51] and POLA1 [52] have been reported as targets for anti-cancer therapy. The known functions of the selected hub proteins are thus consistent with our data about the anti-cancer effects of H-CM. Furthermore, the 8 down-regulated hub proteins were categorized to the GO terms of leading strand elongation, DNA replication, DNA strand elongation involved in DNA replication, and regulation of transcription involved in the G1/S transition of the mitotic cell cycle (Figure 4E, Table 2). The KEGG pathway analysis returned only one term from the down-regulated hub proteins, hsa05166:HTLV-I infection (Figure 4F, Table 2), which was previously connected to tumorigenesis [41]. The GO terms and KEGG pathway also support ours in vitro results showing the anti-cancer effects of H-CM. Therefore, the hub proteins in HeLa cells treated with H-CM could be effective targets for anti-cancer therapies.

H-CM treatment showed anti-cancer effects in other cervical cancer cells (CaSki, ME-180, and SiHa cells), melanoma cells (A375SM), breast cancer cells (MCF-7), and lung cancer cells (NCI-H358) (Supplementary Figure S3A–F). Thus, H-CM has strong anti-cancer effects on multiple types of cancer cells. The effects of LTBR treatment and the hub proteins in those cancer cells related to the anti-cancer effects of H-CM will be investigated in future studies.

In contrast to our results showing that CM from hypoxic HDFs has anti-cancer effects, other studies have shown that CAFs support tumor growth [53] and that hypoxic conditions induce CAFs to secrete TGF-β2, which promotes chemoresistance in cancer cells [54]. Therefore, CAFs have been researched as a target for anti-cancer therapy [55]. The contradictory effects and mechanisms of hypoxic HDFs and CAFs on cancer cells will be investigated in future studies. In addition, to evaluate the efficacy and safety of hypoxic-cultured HDFs in vivo, future preclinical experiments will be performed in which hypoxic HDFs, CAFs, and anti-cancer proteins from H-CM will be injected into immunodeficient mice with tumors.

## 4. Materials and Methods

### 4.1. Cell Culture

HDFs (PromoCell GmbH, Heidelberg, Germany) and HeLa cells (ATCC, Manassas, VA, USA) were cultured in Dulbecco’s modified Eagle medium (DMEM) supplemented with 10% fetal bovine serum (FBS, Gibco, Grand Island, NY, USA) and 0.1% antibiotics (Gibco) at 37℃ in a 5% CO_2_ incubator (APM-30D; ASTEC, Fukuoka, Japan) [33]. Oxygen levels of 21%, as the normoxic condition, and 1%, as the hypoxic condition, were used for culturing HDFs from passage 4 to passage 6 [33]. When the cell confluency of all cells reached 90%, the cells were passaged using 0.25% Trypsin-EDTA (Gibco).

For the proliferation assay, 2 × 10^5^ HDFs at passage 6 were cultured in a 100-mm culture plate for 5 days, and cell numbers were measured using trypan blue 0.5% solution staining (Biowest, Riverside, MO, USA). Other cervical cancer cells (CaSki, ME-180, and SiHa cells), melanoma cells (A375SM), breast cancer cells (MCF-7), and lung cancer cells (NCI-H358) were purchased from the Korea Cell Line Bank (Seoul, Korea). CaSki, ME-180, A375SM, MCF-7, and NCI-H358 were cultured in RPMI1640 (Gibco) supplemented with 10% FBS (Gibco), 1% L-glutamine (Gibco), 25 mM HEPES (Gibco), 25 mM NaHCO_3_ (Gibco), and 0.1% antibiotics (Gibco). SiHa cells were cultured in DMEM supplemented with 10% FBS (Gibco) and 0.1% antibiotics (Gibco).

### 4.2. Preparation of CM from Normoxic and Hypoxic HDFs

Normoxic and hypoxic HDFs (2 × 10^5^ cells) at passage 6 were cultured in 100-mm culture plates with complete medium. When cell confluency reached 90% on day 5, the medium was removed, and 1× phosphate-buffered saline (PBS) was added to wash the cells. Next, 6 mL of DMEM without FBS or antibiotics were added to the cells. After incubation for 24 h, the N-CM and H-CM were harvested and centrifuged at 300× *g* for 5 min. The supernatant was transferred to new 15-mL tubes and stored at −80 °C. DMEM without FBS or antibiotics was used as the C-CM [33]. To generate concentrated CM with proteins, 6 mL of CM were placed in an Amicon Ultra-15 centrifugal filter with a cut-off of 3000 Dalton (Millipore, Billerica, MA, USA) and centrifuged at 4000× *g* for 1 h. Non-filtered CM and concentrated CM with the proteins in the filter were harvested. The volume of the concentrated CM with proteins was approximately 300 µL from 6 mL of CM. To exclude anti-cancer effects caused by exhausted metabolites in the CM, the concentrated CM with proteins (300 µL) was mixed with fresh serum-free medium (5.7 mL) and prepared in the same volume (6 mL, 1:20 dilution) as the non-filtered CM.

### 4.3. Cell Viability Assay

Normoxic and hypoxic HDFs (1 × 10^3^ cells) at passage 6 were seeded in 96-well plates. After 5 days of culture, 100 µL of CellTiter-Glo assay 2.0 reagent (Promega, Madison, WI, USA) were applied to the cells. After 10 min of incubation, the luminescence ratio indicating cell viability was measured using a GLOMAX Multi Detection System (Promega Biosystems Sunnyvale, CA, USA) [33]. To analyze the viability of HeLa, CaSki, ME-180, and SiHa cells treated with CM from HDFs, 1 × 10^4^ cells were seeded in 96-well plates. The culture medium was removed the following day, and 100 µL of CM were applied to the cells. To confirm the anti-cancer effects of the proteins in the CM, 100 µL of non-filtered CM, concentrated CM with proteins, and concentrated CM with proteins mixed with fresh serum-free medium were administered to HeLa cells. Recombinant LTBR protein (Euprotein Inc., North Brunswick, NJ, USA) was administered to HeLa cells at various doses (0, 125, 250, 500, 1000, 2000 ng/mL) for 72 h. Cell viability was assessed using CellTiter-Glo assay 2.0 reagent (Promega) as described [33].

### 4.4. Apoptosis Assay

HeLa cells (1.5 × 10^5^) were seeded in 6-well culture plates [33]. The next day, the culture medium was removed, and the cells were treated with 2 mL of C-CM, N-CM, or H-CM. After 48 h of incubation, the cells were harvested with 0.25% Trypsin-EDTA (Gibco) and stained using a Fluorescein Isothiocyanate Annexin-V Apoptosis Detection Kit I (BD Pharmingen, San Diego, CA, USA). The stained cells were analyzed with a flow cytometer (Becton-Dickinson, San Jose, CA, USA) and FlowJo v10 (Treestar) [33].

### 4.5. Caspase 3/7 Activity Assay

HeLa cells (1 × 10^4^) were seeded in 96-well plates [33]. The next day, the culture medium was removed, and 100 µL of C-CM, N-CM, or H-CM were applied to the cells. After 12, 24, or 48 h of incubation, 100 µL of Caspase-Glo 3/7 Assay reagent (Promega) were added to the cells, and samples were incubated for 1 h. The luminescence ratio indicating caspase 3/7 activity was analyzed using a GLOMAX Multi Detection System (Promega Biosystems Sunnyvale) [33].

### 4.6. Mitochondrial Membrane Potential Assay

HeLa cells (1 × 10^4^) were seeded in 96-well plates [33]. The culture medium was removed the next day, and 100 µL of C-CM, N-CM, or H-CM were applied to the cells. After 12, 24, or 48 h of incubation, the cells were stained using an Orange Mitochondrial Membrane Potential Assay Kit (Abcam, Cambridge, UK). The fluorescence ratio (Ex/Em = 540/590 nm) indicating MMP was measured using a Mithras2 LB 943 Multimode Reader (Berthold Biotechnologies, Bad Wildbad, Germany) [33].

### 4.7. Cell Cycle Assay

HeLa cells (1.5 × 10^5^) were seeded in 6-well culture plates [33]. After an overnight incubation, the culture medium was removed, and cells were treated with 2 mL of C-CM, N-CM, or H-CM. After 24 or 48 h of incubation, the cells were harvested with 0.25% Trypsin-EDTA and fixed with 70% alcohol at 4 °C for 1 h. The fixed cells were stained with 20 μg/mL of propidium iodide (PI; Abcam) and 1% RNase A (Qiagen, Valencia, CA, USA) for 30 min at 37°C. The stained cells were suspended in PBS and analyzed using a FACSVerse flow cytometer (BD Biosciences) and FlowJo v10 (Treestar) [33].

### 4.8. Analysis of Secretory Proteins with Protein Antibody Array

The secretory proteins in N-CM and H-CM were analyzed using a RayBio Label-based (L-Series) Human L1000 Antibody Array (RayBiotech, Inc., Norcross, GA, USA) by E-biogen (Kyung Hee Business Center, Kyung Hee University, Seoul, Korea). Briefly, the array slides were dried for 2 h and incubated with blocking solution at room temperature for 30 min. After removing the blocking buffer, 400 µL of CM were added to the array slides and left for 2 h. After removing the CM, the slides were washed with 1× wash buffer I and 1× wash buffer II. Next, the array slides were treated with 1× biotin-conjugated anti-cytokine antibodies for 2 h at room temperature, and then they were treated with 1× Cy3-conjugated streptavidin stock solution for 2 h. After the slides were washed with 1× wash buffer I for 10 min twice, deionized water was added, and the slides were centrifuged at 1000 rpm for 3 min. Then, the buffer was removed. The slides were scanned using a GenePix 4100A Scanner (Axon Instrument, San Jose, USA), and data were analyzed with GenePix 7.0 (Axon Instrument) and Genowiz 4.0^TM^ (Ocimum Biosolutions, Madhapur, India) [33]. Proteins that were up- or down-regulated (>2-fold) in H-CM compared with N-CM were described using the UniProt DB and MeV v 4.9.0 (J. Craig Venter Institute), and the GO terms and KEGG pathways of the proteins were determined using DAVID (*p* < 0.01) [33]. The data from the protein antibody array are available at the Gene Expression Omnibus (GEO) under the accession numbers GSE185978 and GSE185980.

### 4.9. Analysis of the Intracellular Signaling Pathways Using a Protein Antibody Array

HeLa cells (2 × 10^5^) were cultured in 100-mm culture plates with complete medium. When cell confluency reached 90%, the culture medium was removed, and the cells were washed in 1× PBS. Next, 10 mL of N-CM or H-CM was administered to the cells for 24 h. The cells were harvested, and intracellular proteins were analyzed with a Signaling Explorer antibody array (Full Moon Biosystems, Sunnyvale, CA, USA) by E-biogen (Kyung Hee Business Center) [33]. After extracting protein from the cells using a protein extraction buffer (Full Moon Biosystems) containing a 1% protease inhibitor cocktail (Sigma, St. Louis, MO, USA), 1% phosphatase inhibitor cocktail (Sigma), and lysis beads (Full Moon Biosystems), the protein solution was purified using a gel matrix column (Full Moon Biosystems). The concentration of the purified protein was measured with a BCA protein assay kit (Pierce, Rockford, IL, USA) and NanoPhotometer® (Implen, Westlake Village, USA) Protein samples (50 µg) were treated with biotin/*N*,*N*-dimethylformamide solution for 90 min and stop reagent for 30 min. The Signaling Explorer antibody microarray slides (Full Moon Biosystems) were treated with blocking solution and incubated on a shaker at 60 rpm for 30 min at room temperature. Next, the slides were washed with Milli-Q grade water, and the sample was mixed in coupling solution. The blocked array slides were incubated with coupling mixture for 2 h, and then the slides were washed with washing solution for 5 min. Cy3-streptavidin (GE Healthcare, Chalfont St. Giles, UK) was mixed in 30 mL of detection buffer and administered to the coupled array slides for 20 min. The slides were washed six times with washing solution for 5 min each time and then with Milli-Q grade water. Slide scanning was performed using a GenePix 4100A scanner (Axon Instrument), and data were analyzed using GenePix 7.0 (Axon Instrument) and Genowiz 4.0^TM^ (Ocimum Biosolutions) [33]. Protein information was annotated using the UniProt DB and MeV v 4.9.0 (J. Craig Venter Institute). The GO terms and KEGG pathways of proteins up- or down-regulated were analyzed using DAVID (*p* < 0.01) [33]. PPIs were analyzed using the STRING database and Cytoscape 3.7.0 (NIGMS). The numbers of nodes (proteins) and edges (protein interaction lines) were analyzed, and nodes with more than five edges in a PPI were defined as hub proteins. The data from the protein antibody array are available at GEO under the accession numbers GSE185979 and GSE185980.

### 4.10. ELISA

Six milliliters of N-CM and H-CM were concentrated using an Amicon Ultra-15 centrifugal filter with a cut-off of 3000 Dalton (Millipore, Billerica, MA, USA) at 4000× *g* for 1 h. The concentrated CMs in the filter were harvested. The amount of LTBR in the CMs was measured using a RayBio Human LTBR ELISA Kit (RayBiotech, Norcross, GA, USA) as described in the manufacturer’s manual. Briefly, 100 µL of each concentrated CM were added to a 96-well plate coated with anti-human LTBR antibody (RayBiotech), and the plate was incubated for 2.5 h at room temperature. The antibody solution was discarded, and each well was washed with 1X wash solution (RayBiotech) four times. Then, 100 µL of biotinylated antibody (RayBiotech) were added to each well, and the samples were incubated for 1 h. The biotinylated antibody solution was discarded, and the washing step was repeated. Next, 100 µL of streptavidin solution (RayBiotech) was added to each well, and the samples were incubated for 45 min. The streptavidin solution was discarded, and the washing step was repeated. Next, 100 µL of TMB One-Step substrates reagent (RayBiotech) was added to each well, and the samples were incubated for 30 min at room temperature. Finally, 50 µL of stop solution (RayBiotech) was added to each well. The absorbance of each well was measured using an xMark microplate spectrophotometer (BioRad) at 450 nm.

### 4.11. Western Blotting

HeLa cells treated with N-CM or H-CM for 24 h were harvested and lysed with PRO-PREP lysis buffer (iNtRON Biotechnology, Seongnam, Korea). The proteins in the cell lysate were quantified using Quick Start Bradford 1x dye reagent (Bio-Rad Laboratories, USA). Next, 20 µg of protein lysates were separated by SDS-PAGE and transferred to nitrocellulose membranes (Bio-Rad Laboratories, Germany). The membranes were blocked with 4% skim milk for 2 h at room temperature and then incubated with rabbit anti-human CD19 (1:3000 dilution; cat. no. CSB-RA780821A0HU; CUSABIO, Wuhan, China), CHEK1 (1:3000 dilution; cat. no. CSB-RA176809A0HU; CUSABIO), ESR1 (1:3000 dilution; cat. no. CSB-PA11399A0Rb; CUSABIO), LCK (1:3000 dilution; cat. no. CSB-PA009798; CUSABIO), MCL1 (1:3000 dilution; cat. no. CSB-PA03829A0Rb; CUSABIO), PCNA (1:3000 dilution; cat. no. CSB-PA-208009; CUSABIO), POLA1 (1:3000 dilution; cat. no. CSB-PA002170; CUSABIO), TBP (1:3000 dilution; cat. no. CSB-RA821481A0HU; CUSABIO), or TNF antibody (1:3000 dilution; cat. no. CSB-PA07427A0Rb; CUSABIO) and mouse anti-human ꞵ-actin antibody (1:10,000 dilution; cat. no. A1978; Sigma) overnight at −4 °C. Secondary goat anti-rabbit IgG(H+L) HRP-conjugated antibody (1:5000 dilution; cat. no. CSB-PA489724; CUSABIO, Wuhan, China) or anti-mouse IgG(H+L) HRP-conjugated antibody (1:30,000 dilution; cat. no. A90-116P; Bethyl Laboratories, Montgomery, TX, USA) was administered to the membranes for 2 h at room temperature. The membranes were then washed using 1 × PBS with 0.1% TWEEN 20 (Sigma), stained with a WEST-SAVE Up Western Blot Detection Kit (AbFrontier, Seoul, Korea), and developed using an Automatic X-RAY Film Processor (JPI Healthcare, Seoul, Korea). The quantitative analysis of protein bands was performed using ImageJ 1.53k software (National Institutes of Health, Bethesda, MD, USA).

### 4.12. Statistical Analysis

All experiments were performed at least three times independently in triplicates (*n* = 3). Antibody array experiments were performed one time. All experimental data were analyzed using *t* test and one-way ANOVA. A *p*-value < 0.05 was considered statistically significant. All analyses were carried out using GraphPad Prism version 7.00 (GraphPad Software).

## 5. Conclusions

In conclusion, our study demonstrates that, compared with N-CM, H-CM shows enhanced anti-cancer effects and induces intracellular signaling patterns and hub proteins that have anti-cancer effects on cervical cancer cells. Our results suggest that hypoxic culture conditions for HDFs could be used to identify anti-cancer factors and investigate intracellular target molecules for developing effective anti-cancer therapies.

## Figures and Tables

**Table 1:** Nodes and edges in the PPIs of intracellular proteins up- and down-regulated (>2-fold) in HeLa cells treated with H-CM compared with N-CM.

Node	Number of Edges	Fold Change	SwissProt
TNF	18	3.002 (+)	P01375
ESR1	12	2.142 (−)	P57753
MCL1	7	2.035 (−)	Q07820
TBP	7	2.355 (−)	P20226
CD19	6	2.257 (−)	P15391
LCK	6	2.030 (−)	P06239
PCNA	6	2.172 (−)	P12004
CHEK1	5	2.205 (−)	O14757
POLA1	5	2.022 (−)	P09884
ACTG2	4	2.338 (−)	P63267
APAF1	4	2.327 (−)	O14727
HPRT1	4	2.468 (−)	P00492
PTH	4	2.113 (−)	P01270
REN	4	2.297 (+)	P00797
USP13	4	2.200 (−)	Q92995
BCL10	3	2.018 (−)	O95999
CD8A	3	2.181 (−)	P01732/P10966
GATA1	3	2.055 (−)	P15976
PLK2	3	2.206 (+)	Q9NYY3
ACTR3	2	2.082 (−)	P61158
ADRA2A	2	2.096 (−)	P08913
ANXA6	2	2.433 (−)	P08133
ALDH3B1	2	2.358 (−)	P43353
COL3A1	2	2.315 (+)	P02461
ADGRE1	2	2.042 (−)	Q14246
HMGB1	2	2.070 (−)	P09429
S100A6	2	2.027 (−)	P06703
TFAP2A	2	2.465 (−)	P05549
POU2F2	2	2.073 (−)	P09086
USP19	2	2.089 (−)	O94966
USP30	2	2.140 (−)	Q70CQ3
ADH7	1	2.264 (−)	P40394
DNM1	1	2.071 (-)	Q05193
ELK1	1	2.165 (-)	P19419
GAD1	1	2.075 (+)	Q99259
GPRIN2	1	3.252 (+)	O60269
GPR18	1	2.408 (−)	Q14330
GRTP1	1	3.786 (+)	Q5TC63
IAPP	1	2.501 (−)	P10997
IP6K2	1	2.393 (−)	Q9UHH9
NFE2L2	1	2.217 (−)	Q16236
PTGS1	1	2.427 (+)	P23219
POLL	1	2.090 (−)	Q9UGP5
TNNI3	1	2.246 (−)	P19429
TNXB	1	2.044 (−)	P22105
TUBGCP3	1	2.331 (+)	Q96CW5
USP24	1	2.122 (−)	Q9UPU5

**Table 2:** GO terms and KEGG pathways of hub proteins (≥5 edges) down-regulated (>2-fold) in HeLa cells treated with H-CM compared with N-CM (*p* < 0.01).

DAVID	Category	Term	Protein	−log_10_ (*p*-Value)
GO analysis	Biological process	GO:0006272, leading strand elongation	POLA1, PCNA	2.903
		GO:0006260, DNA replication	POLA1, PCNA, CHEK1	2.763
		GO:0006271, DNA strand elongation involved in DNA replication	POLA1, PCNA	2.205
		GO:0016032, viral process	POLA1, TBP, LCK	2.204
		GO:0000083, regulation of transcription involved in G1/S transition of mitotic cell cycle	POLA1, PCNA	2.020
KEGG pathway analysis	hsa05166:HTLV-I infection		PCNA, TBP, LCK, CHEK1	2.807

## Data Availability

Publicly available datasets were analyzed in this study and can be found here: Gene Expression Omnibus (GEO, www.ncbi.nlm.nih.gov/geo/, accessed on 22 October 2021), GSE185978, GSE185979, GSE185980.

## Supplementary Materials

The following supporting information can be downloaded at: https://www.mdpi.com/article/10.3390/ijms23095134/s1.
