# Supplementary material for: Enhanced Anti-Cancer Effects of Conditioned Medium from Hypoxic Human Adult Dermal Fibroblasts on Cervical Cancer Cells
Source: Int J Mol Sci. 2022 May 4;23(9):5134. doi: 10.3390/ijms23095134 (PMC9100075; doi:10.3390/ijms23095134)
Supplement: Supplementary file 1 [file ijms-23-05134-s001.zip › Supplementary Figures.pdf]

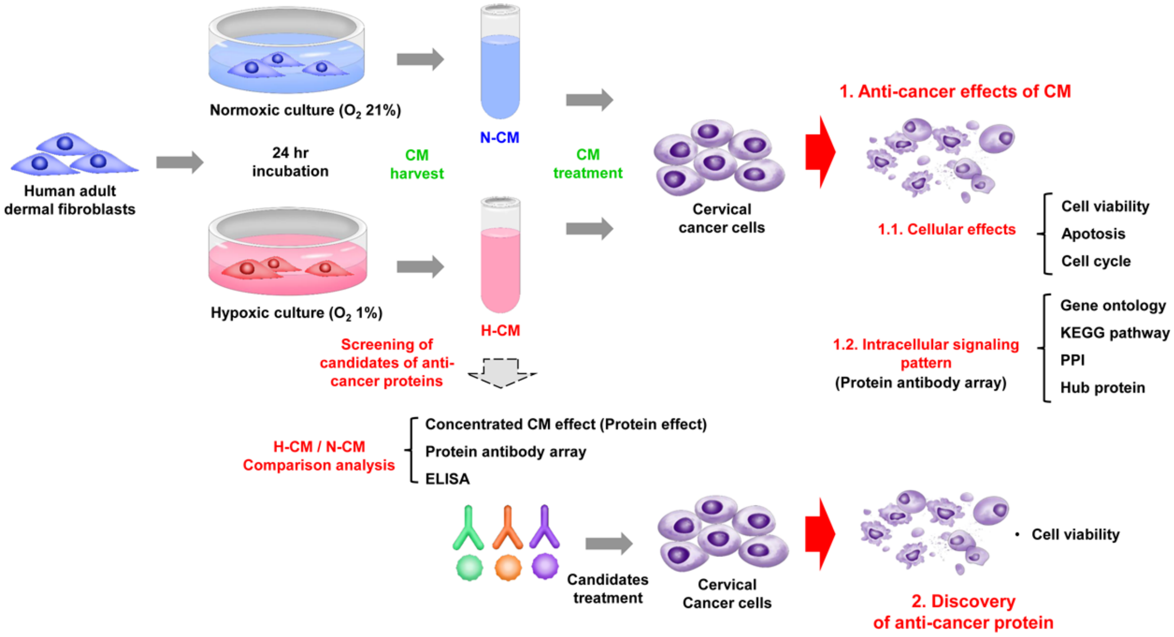

Figure S1. A graphical representation of study design.

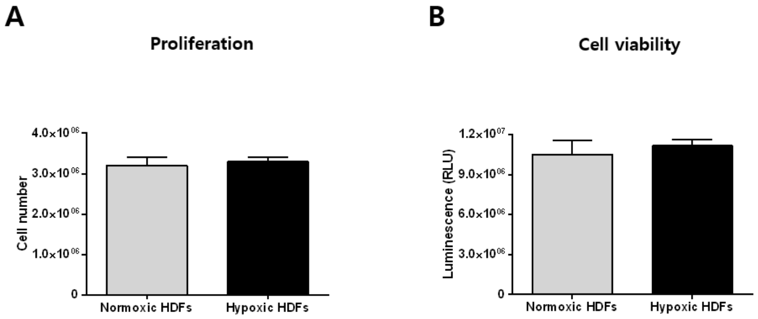

Figure S2. Cell viability of normoxic and hypoxic fibroblasts. Cell proliferation and cell viability of normoxic and hypoxic HDFs after 5 days of culture (A, B). The cells were cultured in normoxic and hypoxic culture conditions for 5 days. Cell proliferation was measured using the trypan blue staining assay, and cell viability was measured using the CellTiter-Glo assay. Data are expressed as the mean ± SD of experiments performed in triplicate (each group n = 3, *p < 0.05, **p < 0.01, t-test). HDFs, human adult dermal fibroblasts. Data images were produced using GraphPad Prism version 7.00 (GraphPad Software).

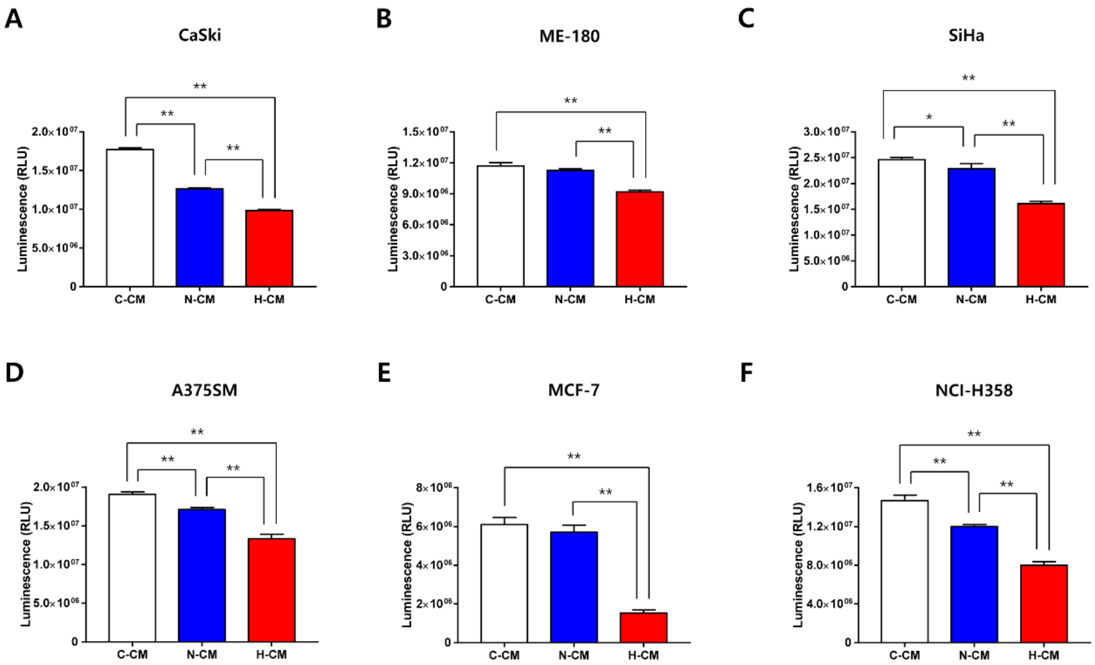

Figure S3. The viability of cervical cancer cells, melanoma cells, breast cancer cells, and lung cancer cells. The viability of three cervical cancer cell lines, CaSki (A), ME-180 (B), and SiHa cells (C), melanoma cells (A375SM) (D), breast cancer cells (MCF-7) (E), and lung cancer cells (NCI-H358) (F) after treatment with H-CM for 72 h, compared with C-CM and N-CM treatment. Cell viability was measured using the CellTiter-Glo assay. Data are expressed as the mean ± SD of experiments performed in triplicate (each group n = 3, *p < 0.05, **p < 0.01, one-way ANOVA). C-CM, serum-free medium as control; N-CM, conditioned medium from normoxic HDFs; H-CM, conditioned medium from hypoxic HDFs. Data images were produced using GraphPad Prism version 7.00 (GraphPad Software).

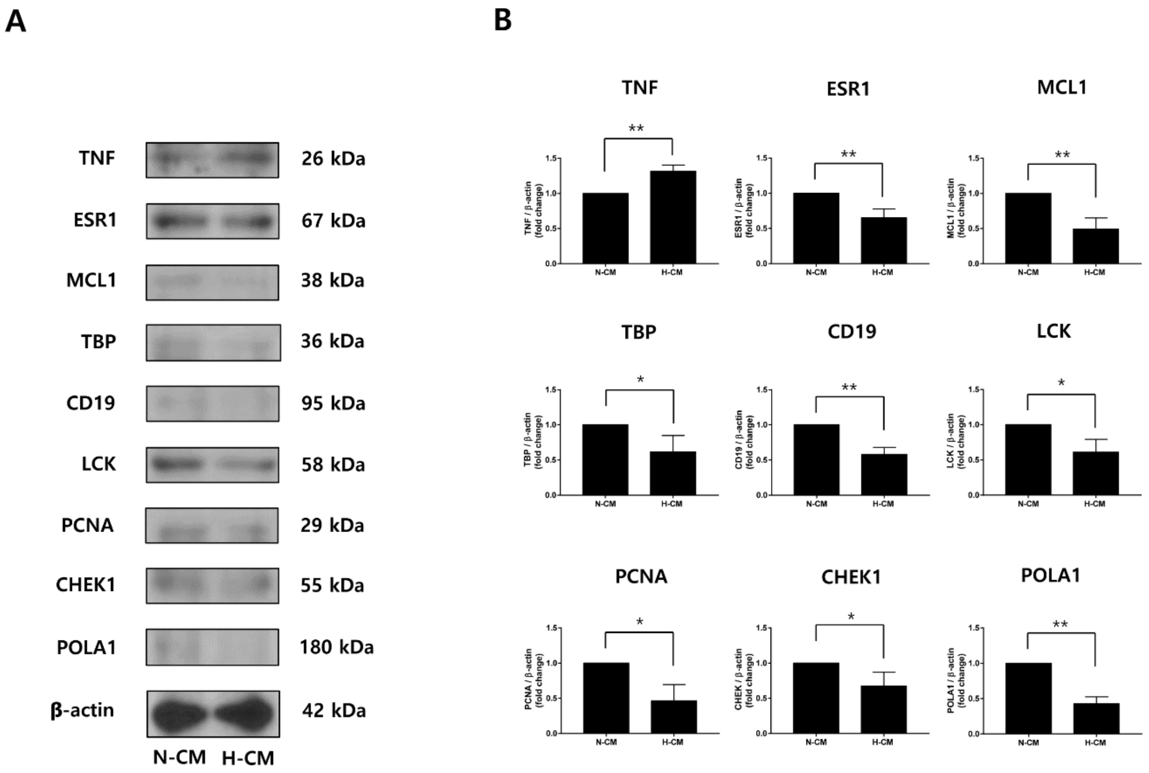

Figure S4. The expression of hub proteins (TNF, ESR1, MCL1, TBP, CD19, LCK, PCNA, CHEK1, and POLA1) in HeLa cells treated with N-CM and H-CM. The expression of hub proteins in HeLa cells treated with N-CM and H-CM for 24 h was analyzed with Western blotting (A). Quantitative amount of each hub proteins in HeLa cells treated H-CM compared with N-CM (B). N-CM, conditioned medium from normoxic HDFs; H-CM, conditioned medium from hypoxic HDFs. Data are expressed as the mean ± SD of three independent experiment (biological replicates n = 3, $^{*}p < 0.05$, $^{**}p < 0.01$, t-test). Data images were produced using GraphPad Prism version 7.00 (GraphPad Software) and ImageJ 1.53k software (National Institutes of Health).
