# Supplementary material for: Enhanced Anti-Cancer Effects of Conditioned Medium from Hypoxic Human Adult Dermal Fibroblasts on Cervical Cancer Cells
Source: Int J Mol Sci. 2022 May 4;23(9):5134. doi: 10.3390/ijms23095134 (PMC9100075; doi:10.3390/ijms23095134)
Supplement: Supplementary file 1 [file ijms-23-05134-s001.zip › Supplementary Tables.pdf]

Table S1. Proteins up- and down-regulated in H-CM compared with N-CM (>2-fold).

Protein	Fold change	SwissProt
LTBR	6.189 (+)	P36941
FGF10	3.507 (+)	O15520
VEGFA	3.236 (+)	P15692
LYVE1	2.568 (+)	Q9Y5Y7
MMP14	2.493 (+)	P50281
TNFRSF10C	2.429 (+)	O14798
IL2RG	2.411 (+)	P31785
TGFBR2	2.407 (+)	P37173
CX3CL1	2.217 (+)	P78423
DKKL1	2.207 (+)	Q9UK85
CCL4	2.200 (+)	P13236
CCL18	2.164 (+)	P55774
SLC2A3	2.150 (+)	P11169
IL37	2.144 (+)	Q9NZH6
LECT2	2.139 (+)	O14960
TNFSF15	2.120 (+)	O95150
NID1	2.120 (+)	P14543
CXCL12	2.077 (+)	P48061
IL17C	2.056 (+)	Q9P0M4
KIT	2.032 (+)	P10721
TIMP2	2.143 (−)	P16035
GRN	2.158 (−)	P28799
FGA	2.191 (−)	P02671
UBB	2.481 (−)	P0CG47
F13A1	2.737 (−)	P00488
THBS1	2.843 (−)	P07996
PTHLH	3.365 (−)	P12272
FN1	3.498 (−)	P02751
TNFRSF1A	3.822 (−)	P19438
EDA	9.549 (−)	Q92838
MMP1	11.116 (−)	P03956
PLG	14.235 (−)	P00747

Table S2. GO terms for proteins up-regulated in H-CM compared with N-CM ($p < 0.01$).

DAVID	Category	Term	Protein	$-\log_{10}$ (p-value)
GO analysis	Biological process	GO:0006955, immune response	TNFRSF10C, LTBR, TNFSF15, IL2RG, CX3CL1, CXCL12, CCL4, CCL18, IL37	8.062
		GO:0006954, inflammatory response	IL17C, TNFRSF10C, LTBR, KIT, CXCL12, CCL4, CCL18, IL37	6.948
		GO:0007165,signal transduction	TNFRSF10C, LYVE1, LTBR, TNFSF15, IL2RG, KIT, DKKL1, CXCL12, CCL4, CCL18	5.766
		GO:0070374, positive regulation of ERK1 and ERK2 cascade	VEGFA, FGF10, CX3CL1, CCL4, CCL18	4.408
		GO:0070098, chemokine-mediated signaling pathway	CX3CL1, CXCL12, CCL4, CCL18	4.175
		GO:0050930, induction of positive chemotaxis	VEGFA, FGF10, CXCL12	3.899
		GO:0048754, branching morphogenesis of an epithelial tube	VEGFA, FGF10, MMP14	3.520
		GO:0043547, positive regulation of GTPase activity	FGF10, IL2RG, CX3CL1, KIT, CCL4, CCL18	3.478
		GO:0006935, chemotaxis	CX3CL1, LECT2, CXCL12, CCL18	3.478
		GO:0050918, positive chemotaxis	VEGFA, FGF10, CXCL12	3.152
		GO:0001666, response to hypoxia	VEGFA, TGFBR2, MMP14, CXCL12	3.043
		GO:0002548, monocyte chemotaxis	CX3CL1, CCL4, CCL18	2.993
		GO:0001541, ovarian follicle development	VEGFA, KIT, MMP14	2.993
		GO:0042127, regulation of cell proliferation	TNFRSF10C, LTBR, TGFBR2, KIT	2.951
		GO:0071346, cellular response to interferon-gamma	CX3CL1, CCL4, CCL18	2.729
		GO:0009612, response to mechanical stimulus	TGFBR2, MMP14, CXCL12	2.700
		GO:0060326, cell chemotaxis	CX3CL1, KIT, CXCL12	2.617
		GO:0030593, neutrophil chemotaxis	CX3CL1, CCL4, CCL18	2.603
		GO:0071347, cellular response to interleukin-1	CX3CL1, CCL4, CCL18	2.541
		GO:0050729, positive regulation of inflammatory response	CX3CL1, CCL4, CCL18	2.517
		GO:0071356, cellular response to tumor necrosis factor	CX3CL1, CCL4, CCL18	2.170
		GO:0045766, positive regulation of angiogenesis	VEGFA, TGFBR2, CX3CL1	2.133
		GO:0033209, tumor necrosis factor–mediated signaling pathway	TNFRSF10C, LTBR, TNFSF15	2.111
		GO:0048842, positive regulation of axon extension involved in axon guidance	VEGFA, CXCL12	2.103

		GO:0019221, cytokine-mediated signaling pathway	CX3CL1, KIT, IL37	2.024

Table S3. GO terms of proteins down-regulated in H-CM compared with N-CM ($p < 0.01$).

DAVID	Category	Term	Protein	$-\log_{10}$ (p-value)
GO analysis	Biological process	GO:0002576, platelet degranulation	FGA, F13A1, THBS1, PLG, FN1	6.370
		GO:0022617, extracellular matrix disassembly	TIMP2, PLG, MMP1, FN1	4.844
		GO:0044267, cellular protein metabolic process	FGA, UBB, PLG, MMP1	4.271
		GO:0018149, peptide cross-linking	F13A1, THBS1, FN1	3.328
		GO:0033209, tumor necrosis factor–mediated signaling pathway	TNFRSF1A, UBB, EDA	2.588
		GO:0007596, blood coagulation	FGA, F13A1, PLG	2.211
		GO:0051918, negative regulation of fibrinolysis	THBS1, PLG	2.185
		GO:0030198, extracellular matrix organization	FGA, THBS1, FN1	2.158

Table S4. Intracellular proteins up- and down-regulated in HeLa cells treated with H-CM compared with N-CM (>2-fold).

Protein	Fold change	SwissProt
SLC6A16	5.235 (+)	Q9GZN6
GRTP1	3.786 (+)	Q5TC63
GPRIN2	3.252 (+)	O60269
TNF	3.002 (+)	P01375
SLU7	2.928 (+)	O95391
RAB40B	2.797 (+)	Q12829
CLDN6	2.719 (+)	P56747
UBR1	2.578 (+)	Q8IWV7
KRT15	2.573 (+)	P19012
RAB11FIP4	2.558 (+)	Q86YS3
PTGS1	2.427 (+)	P23219
ACAD10	2.402 (+)	Q6JQN1
TUBGCP3	2.331 (+)	Q96CW5
COL3A1	2.315 (+)	P02461
F12	2.300 (+)	P00748
REN	2.297 (+)	P00797
PLK2	2.206 (+)	Q9NYY3
GAD1	2.075 (+)	Q99259
ACVR1C	2.058 (+)	Q8NER5
SERPINB9	2.006 (+)	P50453
SPTBN5	2.002 (−)	Q9NRC6
SLC4A10	2.005 (−)	Q6U841
BCL10	2.018 (−)	O95999
LILRA1	2.020 (−)	O75019
FES	2.022 (−)	P07332
POLA1	2.022 (−)	P09884
S100A6	2.027 (−)	P06703
LCK	2.030 (−)	P06239
MCL1	2.035 (−)	Q07820
ADGRE1	2.042 (−)	Q14246
TNXB	2.044 (−)	P22105
GATA1	2.055 (−)	P15976
MRC2	2.069 (−)	Q9UBG0
HMGB1	2.070 (−)	P09429
SLC25A6	2.070 (−)	P12236
DNM1	2.071 (−)	Q05193
POU2F2	2.073 (−)	P09086
SVOP	2.081 (−)	Q8N4V2
ACTR3	2.082 (−)	P61158
SLC24A4	2.087 (−)	Q8NFF2
USP19	2.089 (−)	O94966
POLL	2.090 (−)	Q9UGP5
ADRA2A	2.096 (−)	P08913
SLC28A2	2.098 (−)	O43868
PTH	2.113 (−)	P01270
LDLRAD1	2.118 (−)	Q5T700
USP24	2.122 (−)	Q9UPU5

HSFX1	2.137 (−)	Q9UBD0
USP30	2.140 (−)	Q70CQ3
ESR1	2.142 (−)	P57753
ELK1	2.165 (−)	P19419
PCNA	2.172 (−)	P12004
CD8A	2.181 (−)	P01732/P10966
ADGRE2	2.182 (−)	Q9UHX3
USP13	2.200 (−)	Q92995
CHEK1	2.205 (−)	O14757
NFE2L2	2.217 (−)	Q16236
TNNI3	2.246 (−)	P19429
PRKN	2.254 (−)	O60260
CD19	2.257 (−)	P15391
ADH7	2.264 (−)	P40394
APAF1	2.327 (−)	O14727
SLK	2.328 (−)	Q9H2G2
ACTG2	2.338 (−)	P63267
TBP	2.355 (−)	P20226
ALDH3B1	2.358 (−)	P43353
IP6K2	2.393 (−)	Q9UHH9
GPR18	2.408 (−)	Q14330
SLC5A3	2.416 (−)	P53794
ANXA6	2.433 (−)	P08133
TFAP2A	2.465 (−)	P05549
HPRT1	2.468 (−)	P00492
TPRA1	2.476 (−)	Q86W33
IAPP	2.501 (−)	P10997
LDLRAD2	2.540 (−)	Q5SZI1
CCNL1	2.596 (−)	Q9UK58

Table S5. GO and KEGG pathways of intracellular proteins down-regulated in HeLa cells treated with H-CM compared with N-CM ($p < 0.01$).

DAVID	Category	Term	Protein	$-\log_{10}$ (p-value)
GO analysis	Biological process	GO:0016579, protein deubiquitination	USP30, USP19, USP24, USP13	2.448
		GO:0042981, regulation of apoptotic process	BCL10, MCL1, SLK, ESR1, APAF1	2.373
		GO:0006366, transcription from RNA polymerase II promoter	GATA1, POU2F2, ESR1, TFAP2A, ELK1, TBP, NFE2L2	2.311
		GO:0006272, leading strand elongation	PCNA, POLA1	2.033
KEGG pathway analysis		hsa05166:HTLV-I infection	SLC25A6, LCK, PCNA, ELK1, CHEK1, TBP	2.422
		hsa03410:base excision repair	POLL, HMGB1, PCNA	2.078
		hsa05340:primary immunodeficiency	CD19, CD8A, LCK	2.053
